# Supplementary material for: Modeling glioblastoma heterogeneity as a dynamic network of cell states
Source: Mol Syst Biol. 2021 Sep 16;17(9):e10105. doi: 10.15252/msb.202010105 (PMC8444284; doi:10.15252/msb.202010105)
Supplement: Supplementary file 6 — Source Data for Figure 5 [file MSB-17-e10105-s004.zip › Figure5A_sourcedata/GSEA_3017/hallmarks_stateE.GseaPreranked.1621934677158/HALLMARK_P53_PATHWAY.html]

Details for gene set HALLMARK\_P53\_PATHWAY[GSEA]

|  || Dataset | state63017 |
| Phenotype | NoPhenotypeAvailable |
| Upregulated in class | na\_pos |
| GeneSet | HALLMARK\_P53\_PATHWAY |
| Enrichment Score (ES) | 0.22632419 |
| Normalized Enrichment Score (NES) | 0.9618577 |
| Nominal p-value | 0.48846152 |
| FDR q-value | 0.5866112 |
| FWER p-Value | 0.842 |
Table: GSEA Results Summary

  

Fig 1: Enrichment plot: HALLMARK\_P53\_PATHWAY      
 Profile of the Running ES Score & Positions of GeneSet Members on the Rank Ordered List

  

| PROBE | GENE SYMBOL | GENE\_TITLE | RANK IN GENE LIST | RANK METRIC SCORE | RUNNING ES | CORE ENRICHMENT || 1 | PDGFA |  |  | 20 | 0.729 | 0.0847 | Yes |
| 2 | S100A10 |  |  | 51 | 0.527 | 0.1291 | Yes |
| 3 | UPP1 |  |  | 78 | 0.438 | 0.1650 | Yes |
| 4 | ATF3 |  |  | 101 | 0.397 | 0.1991 | Yes |
| 5 | PRMT2 |  |  | 152 | 0.333 | 0.1938 | Yes |
| 6 | JUN |  |  | 172 | 0.316 | 0.2194 | Yes |
| 7 | SAT1 |  |  | 206 | 0.291 | 0.2263 | Yes |
| 8 | ZFP36L1 |  |  | 287 | -0.255 | 0.1775 | No |
| 9 | RAD51C |  |  | 333 | -0.264 | 0.1676 | No |
| 10 | NUDT15 |  |  | 356 | -0.270 | 0.1833 | No |
| 11 | ADA |  |  | 371 | -0.273 | 0.2081 | No |
| 12 | TXNIP |  |  | 404 | -0.284 | 0.2150 | No |
| 13 | TPRKB |  |  | 540 | -0.329 | 0.1177 | No |
| 14 | CCP110 |  |  | 636 | -0.381 | 0.0710 | No |
| 15 | BTG1 |  |  | 772 | -0.520 | 0.0016 | No |
| 16 | TM4SF1 |  |  | 823 | -0.619 | 0.0380 | No |
| 17 | PCNA |  |  | 832 | -0.640 | 0.1226 | No |
Table: GSEA details [plain text format]

  

Fig 2: HALLMARK\_P53\_PATHWAY: Random ES distribution      
 Gene set null distribution of ES for **HALLMARK\_P53\_PATHWAY**
